# Supplementary material for: Bayesian refinement of protein structures and ensembles against SAXS data using molecular dynamics
Source: PLoS Comput Biol. 2017 Oct 18;13(10):e1005800. doi: 10.1371/journal.pcbi.1005800 (PMC5662244; doi:10.1371/journal.pcbi.1005800)
Supplement: S1 Archive — (BZ2) [file pcbi.1005800.s011.tar.bz2 › gromacs_4.62_bayesian_saxs_refi/share/html/online/tex.html]

tex file format


## tex file format

|  |  |
| --- | --- |
| Main Table of Contents | **VERSION 4.6** |
| GROMACS homepage | **Sat 19 Jan 2013** |

---

### Description

We use **LaTeX** for *document* processing.
Although the input is not so
user friendly, it has some advantages over *word* processors.

- **LaTeX** knows a lot about formatting, probably much more than you.- The input is clear, you always know what you are doing- It makes anything from letters to a thesis- Much more...

---

http://www.gromacs.org
